# Supplementary material for: Prime editor with rational design and AI-driven optimization for reverse editing window and enhanced fidelity
Source: Nat Commun. 2025 Jun 3;16:5144. doi: 10.1038/s41467-025-60495-w (PMC12134370; doi:10.1038/s41467-025-60495-w)
Supplement: Supplementary file 1 — Supplementary Information [file 41467_2025_60495_MOESM1_ESM.pdf]

Supplementary Figure 1

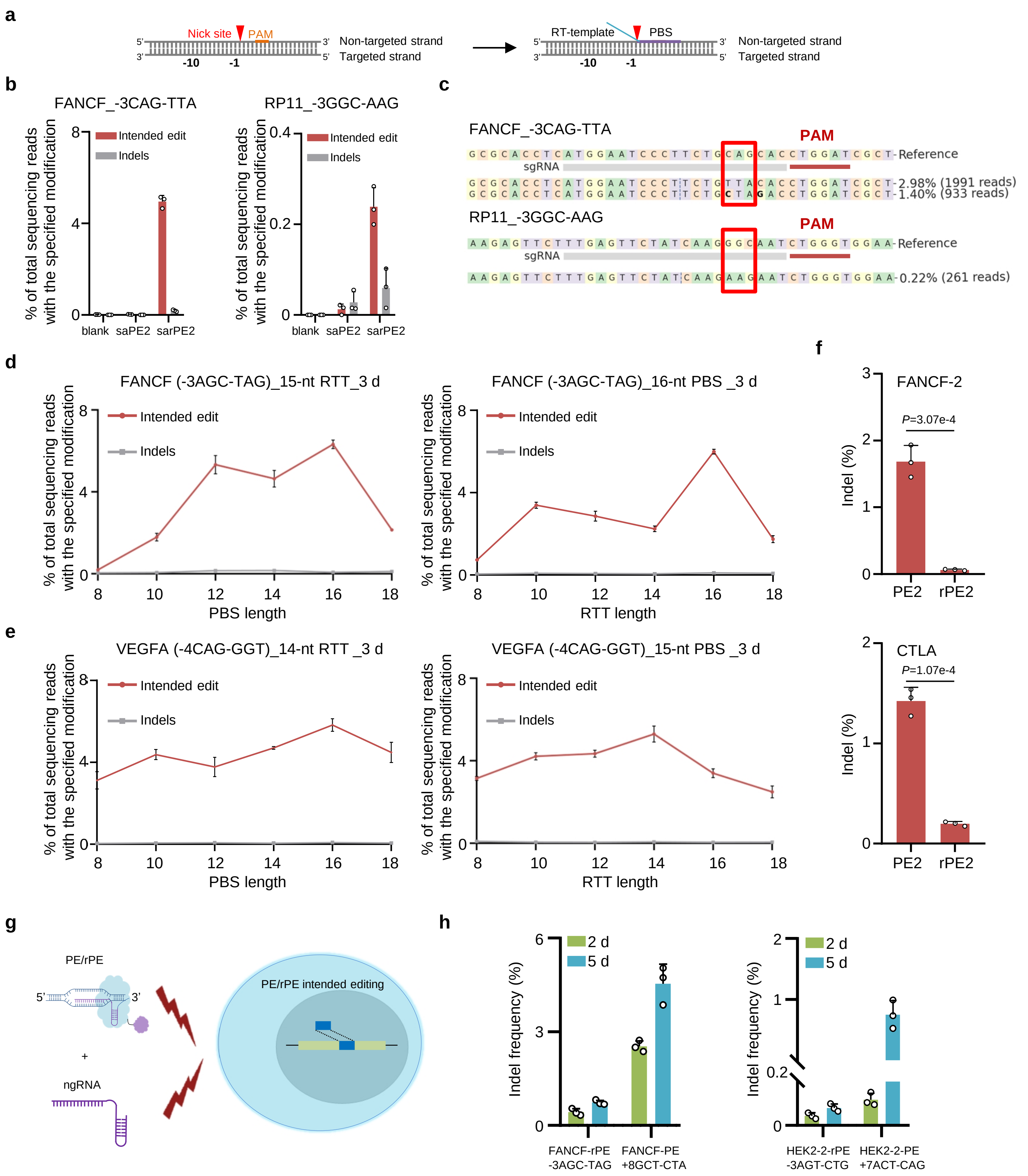

Supplementary Figure 1

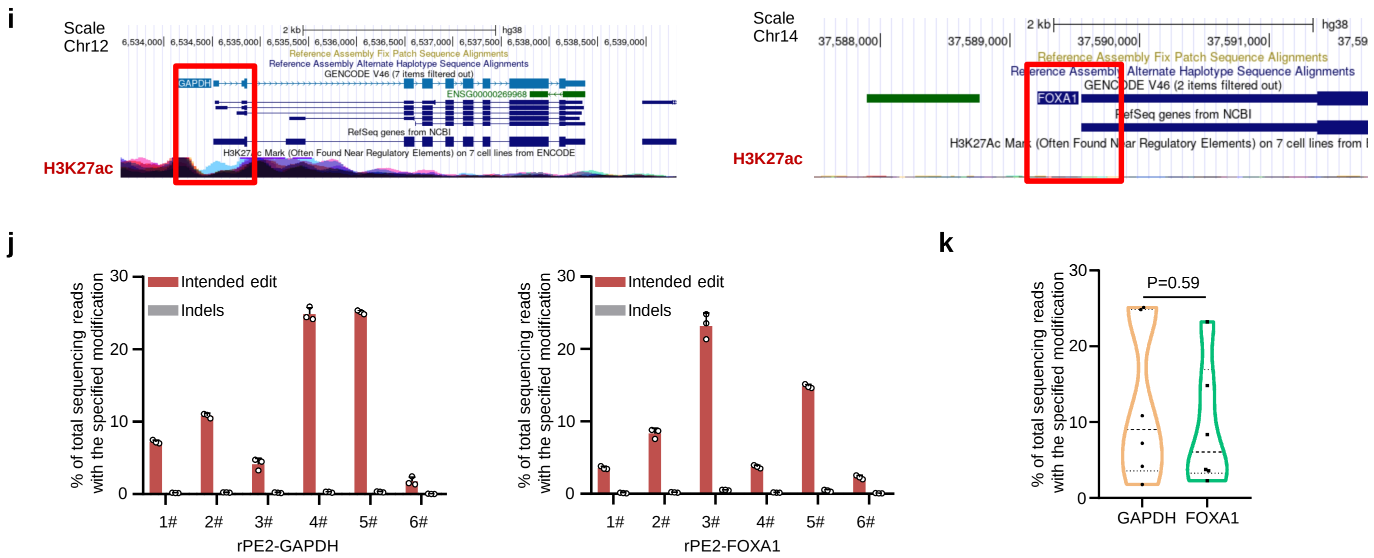

Fig S1. Further data of constructing the rPE

(a) Schematic representation of the reverse prime editing (rPE) strategy. The primer binding site (PBS, purple line) binds to the 3′ direction of the nick site, while the reverse transcription template (RTT) is incorporated in the 5′ direction of the nick site.

(b) Editing efficiency (red bars) and indel frequency (gray bars) at the FANCF and RP11 loci across blank, saPE2, and sarPE2 conditions in HEK293T cells. Data are presented as means ± SD from n = 3 independent biological replicates.

(c) Genotype distribution and editing frequency at the FANCF and RP11 loci for blank, saPE2, and sarPE2 systems in HEK293T cells.

(d–e) Analysis of rPE2 editing efficiency (red lines) and indel frequency (gray lines) across 3 days with different PBS and RTT lengths at the FANCF (d) and VEGFA (e) loci in HEK293T cells. Data are presented as means ± SD from n = 3 independent biological replicates.

(f) Indel frequency comparison between PE2 and rPE2 systems with specified gRNAs targeting the FANCF-2 and CTLA loci. Data are presented as means ± SD from n = 3 independent biological replicates. Statistical significance was assessed using 2-tailed Student’s t-tests.

(g) Schematic showing the detection of indel frequencies between PE and rPE. HEK293T cells were co-transfected with PE/rPE systems, pegRNA/rpegRNA targeting > +6 positions, and a nick gRNA (ngRNA).

(h) Indel frequency at targeted loci in HEK293T cells transfected with PE or rPE systems along with ngRNA at 2- and 5- days post-transfection. Data are presented as means ± SD from n = 3 independent biological replicates.

(i) Schematic of H3K27ac level at *GAPDH* and *FOXA1* promoter.

(j) Editing efficiencies of rPE at 12 genomic loci from *GAPDH* and *FOXA1* promoter in HEK293T cells. Data are presented as means ± SD from n = 3 independent biological replicates.

(k) Average editing efficiencies across these 12 genomic loci between *GAPDH* and *FOXA1* promoter. Data are presented as means ± SD from (n = 6). Statistical significance was assessed using 2-tailed Student’s t-tests.

Supplementary Figure 2

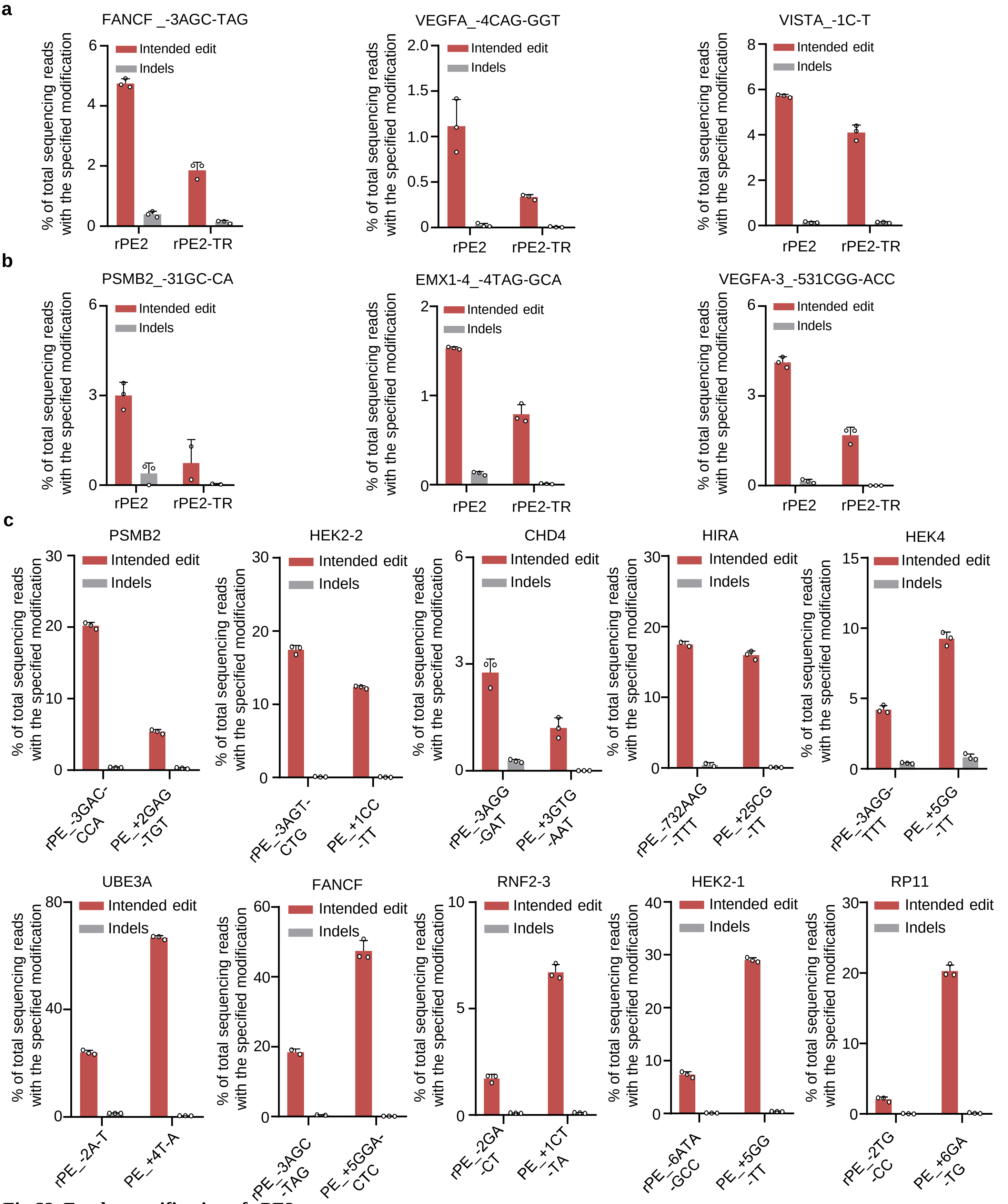

Fig S2. Further verification of rPE2 strategy

(a-b) Editing efficiency (red bars) and indel frequency (gray bars) of rPE2 and rPE2-TR systems across 6 genomic loci in HeLa (a) and HEpG2 (b) cells. Data are presented as means ± SD from n = 3 independent biological replicates.

(c) Comparison of editing efficiency (red bars) and indel frequency (gray bars) between rPE2 and PE2 using the same spacer sequences across 10 genomic loci in HEK293T cells. Data are presented as means ± SD from n = 3 independent biological replicates.

Supplementary Figure 3

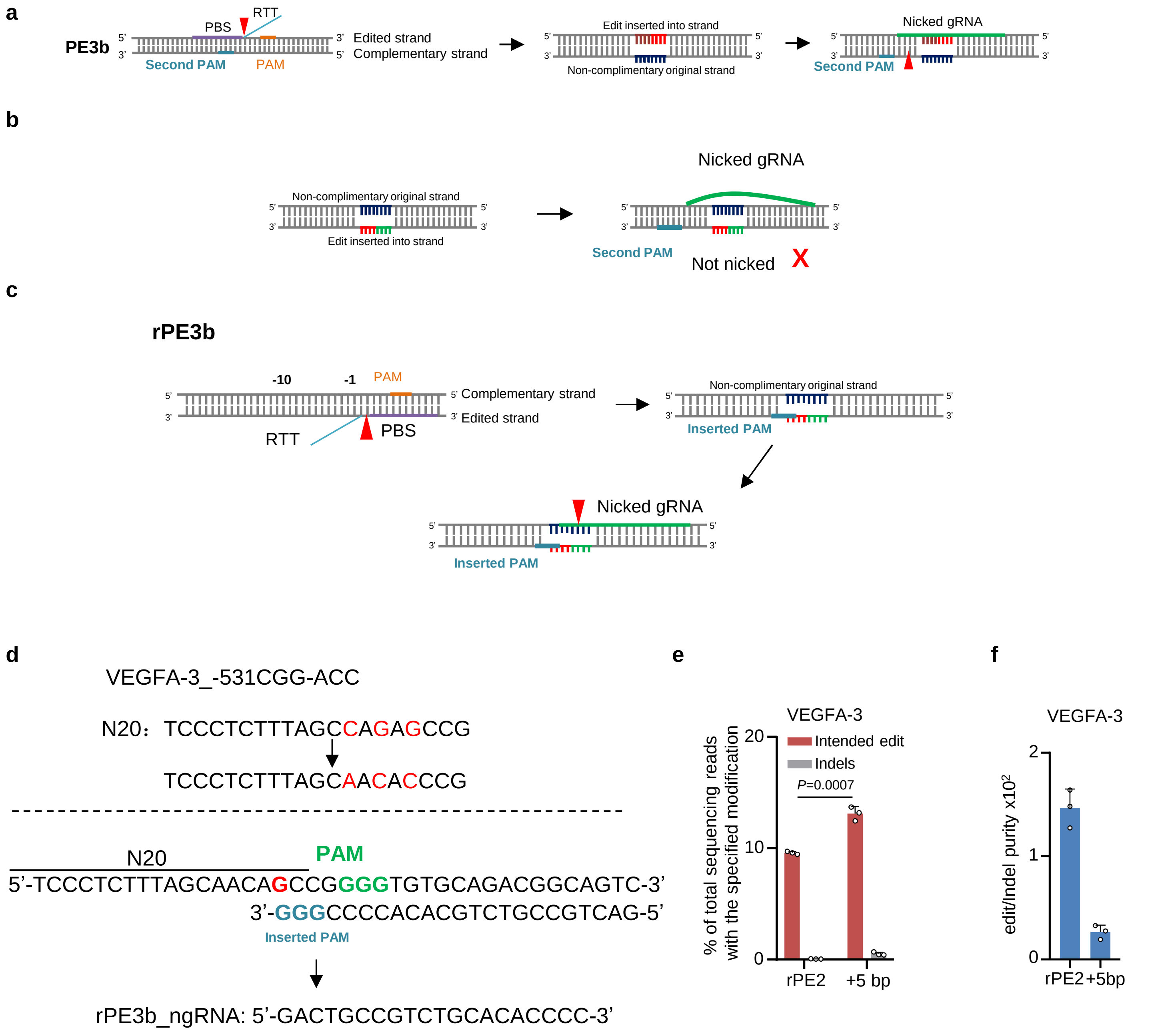

Fig S3. rPE3b strategy and editing efficiency in HEK293T cells

(a) Overview of PE3b strategy. The red arrow indicates the nick site, and the orange line represents the PAM sequence. The nick gRNA is marked with a green line.

(b) Schematic of canonical PE3b strategy for rPE editing. The nick gRNA is marked with a green line.

(c) Overview and schematic of rPE3b strategy. The dark bule represents the inserted PAM, then the ngRNA could only bind to non-targeted strand after the insertion of new PAM.

(d) Schematic of rPE3b editing at VEGFA-3 locus with a new PAM insertion. The dark bule represents the inserted new PAM.

(e) rPE2 and rPE3b editing efficiency (red bars) and indel frequency (gray bars) at VEGFA-3 locus in HEK293T cells. Data are presented as means ± SD from n = 3 independent biological replicates. Statistical significance was assessed using 2-tailed Student’s t-tests.

(f) The editing/indel purity between rPE2 and rPE3b at VEGFA-3 locus in HEK293T cells. Data are presented as means ± SD from n = 3 independent biological replicates.

Supplementary Figure 4

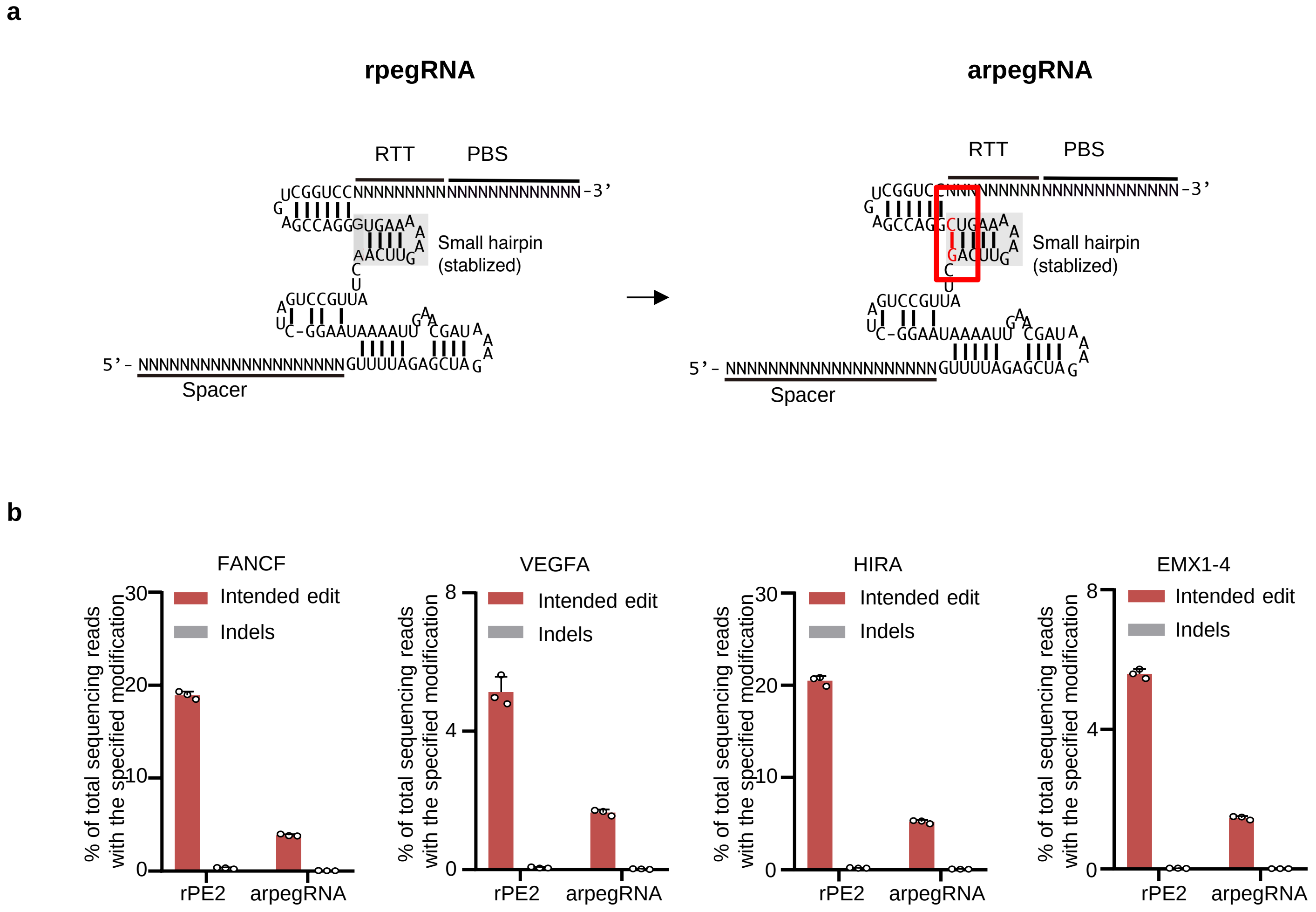

Fig S4. arpegRNA strategy for rPE editing

(a) Schematic of arpegRNA strategy for rPE editing. The modified base pair was marked with red.

(b) rPE2 and rPE2 with arpegRNA editing efficiency (red bars) and indel frequency (gray bars) at 4 genomic loci in HEK293T cells. Data are presented as means ± SD from n = 3 independent biological replicates.

Supplementary Figure 5

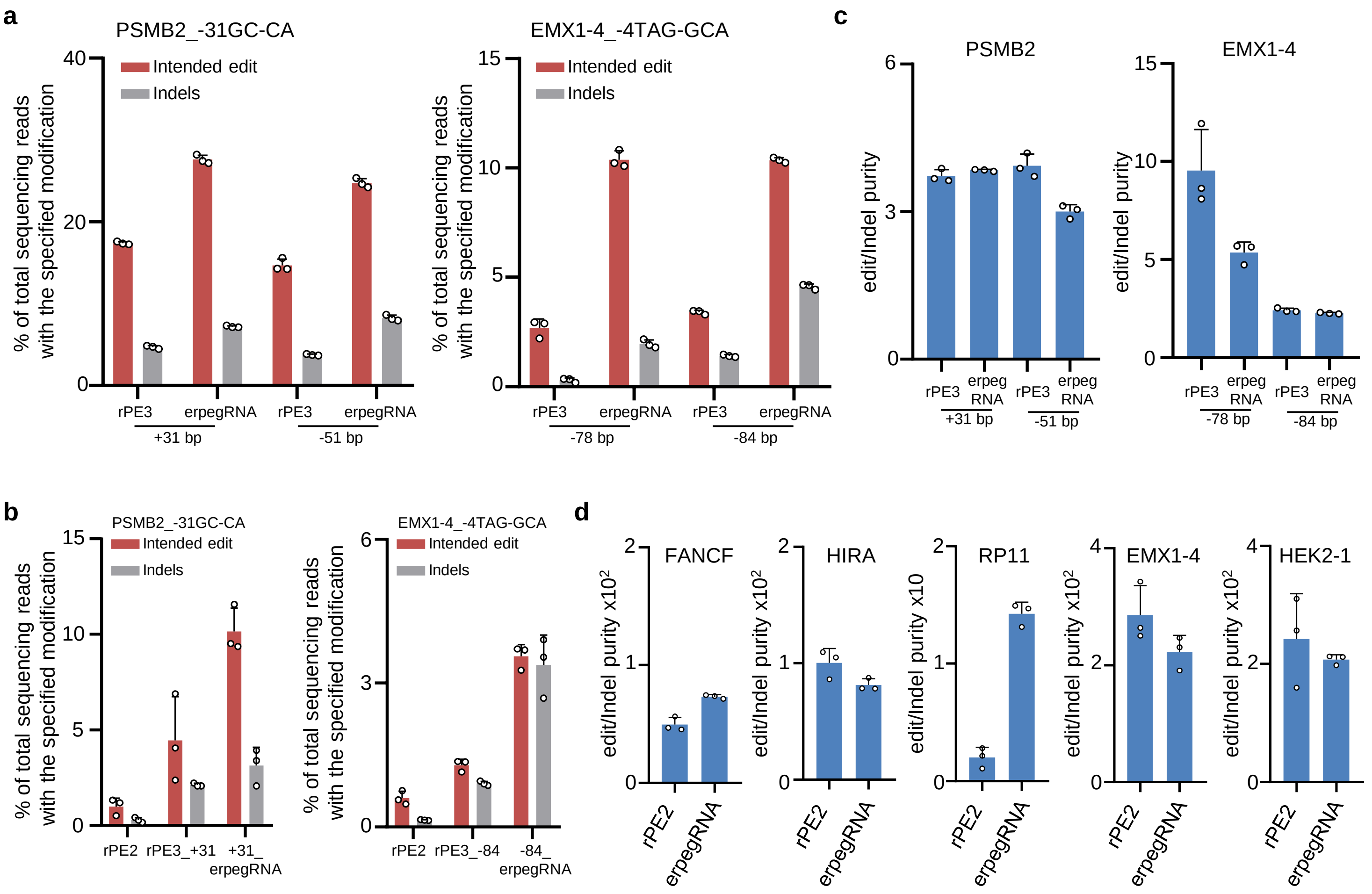

Fig S5. Further data of rPE3 and erpegRNA strategy

(a-b) Editing efficiency (red bars) and indel frequency (gray bars) of rPE2, erpegRNA and together with ngRNA at PSMB2 and EMX1-4 genomic loci in HEK293T (a) and HeLa (b) cells . Data are presented as means ± SD from n = 3 independent biological replicates.

(c) The editing/indel purity across erpegRNA and various ngRNAs at PSMB2 and EMX1-4 genomic loci in HEK293T cells. Data are presented as means ± SD from n = 3 independent biological replicates.

(d) The editing/indel purity between rPE2 and rPE2 with erpegRNA across five genomic loci in HEK293T cells. Data are presented as means ± SD from n = 3 independent biological replicates.

Supplementary Figure 6

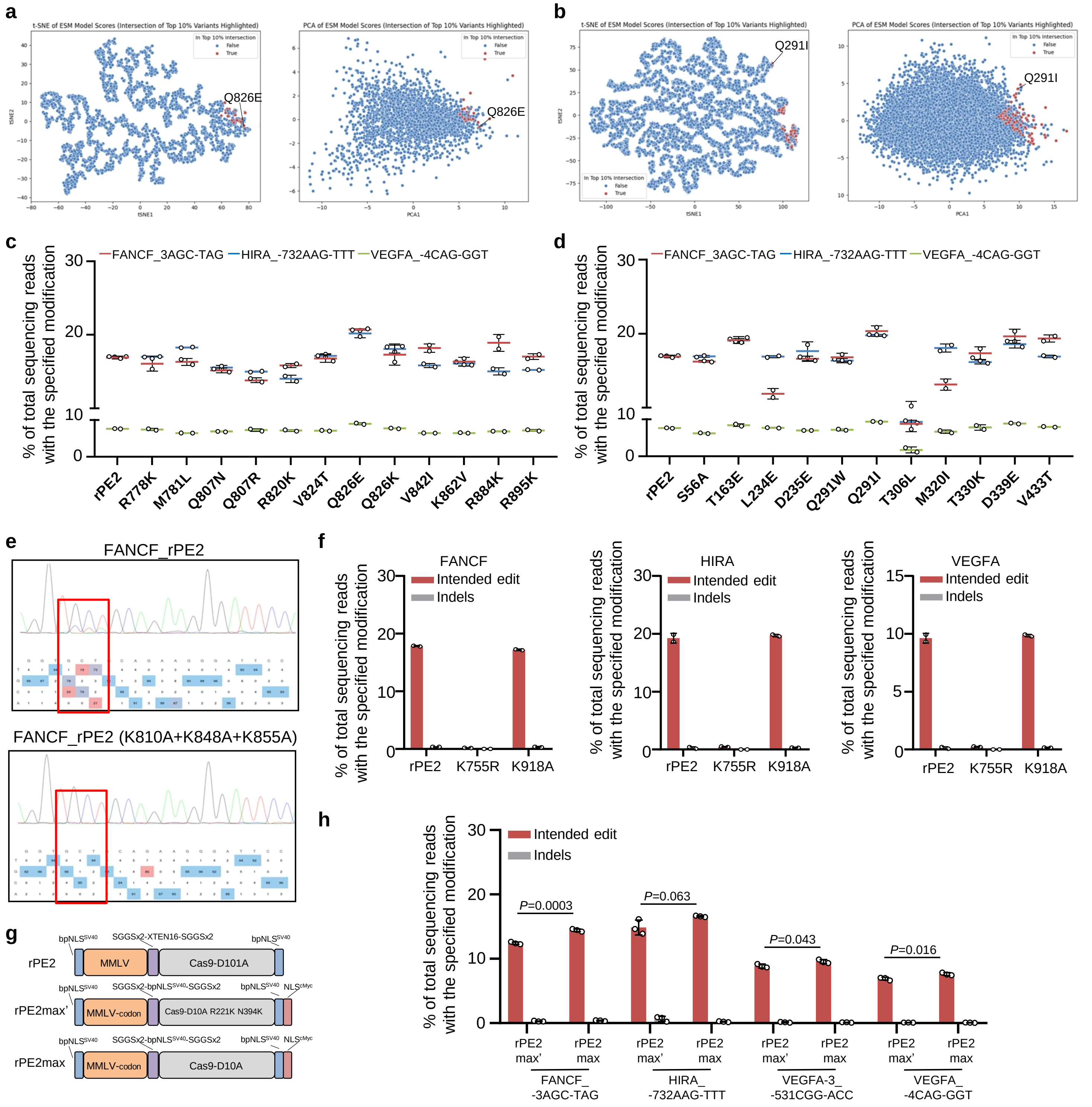

Fig S6. Further verification of erPE2max in human cells

(a-b) Visualization of PCA and t-SNE plots post dimensionality reduction clustering analysis of esm data for HNH (a) and MMLV RT (b). The Q826E in HNH and Q291I in MMLV RT were marked.

(c-d) Editing efficiencies of rPE2 and its evolved variants for HNH (c) and MMLV RT (d) at 3 genomic loci in HKE293T cells. Data are presented as means ± SD from n = 2 independent biological replicates.

(e) Editing efficiency between rPE2 and rPE2 (Cas9-K810A+K848A+K855A) at FANCF locus in HEK293T cells

(f) Editing efficiency (red bars) and indel frequency (gray bars) across rPE2, rPE2_Cas9-K755R and rPE2_Cas9-K918A at FANCF and HIRA genomic loci in HEK293T cells. Data are presented as means ± SD from n = 3 independent biological replicates.

(g) Schematic of rPE2, rPE2max' and rPE2max.

(h) Editing efficiency (red bars) and indel frequency (gray bars) of rPE2max' and rPE2max across 4 genomic loci in HEK293T cells. Data are presented as means ± SD from n = 3 independent biological replicates. Statistical significance was assessed using 2-tailed Student's t-tests.

Supplementary Figure 7

a

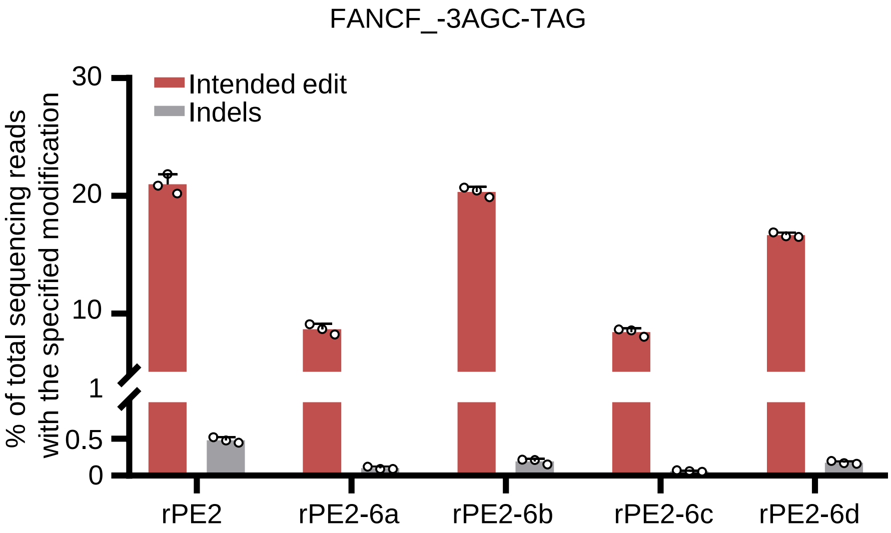

b

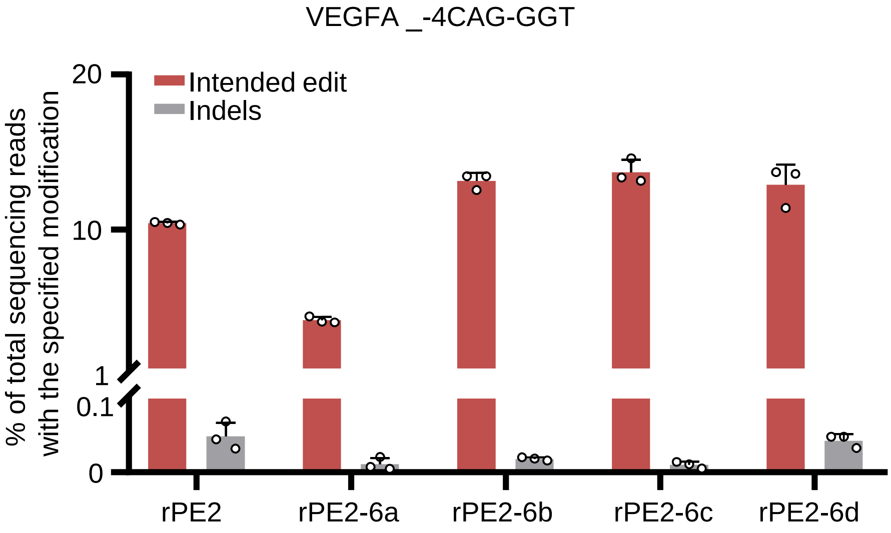

Fig S7. Verification of rPE2 with compact reverse transcriptase

(a-b) Editing efficiency (red bars) and indel frequency (gray bars) across rPE2 and rPE2 with compact reverse transcriptase at FANCF (a) and VEGFA (b) genomic loci in HEK293T cells. Data are presented as means ± SD from n = 3 independent biological replicates.

Supplementary Figure 8

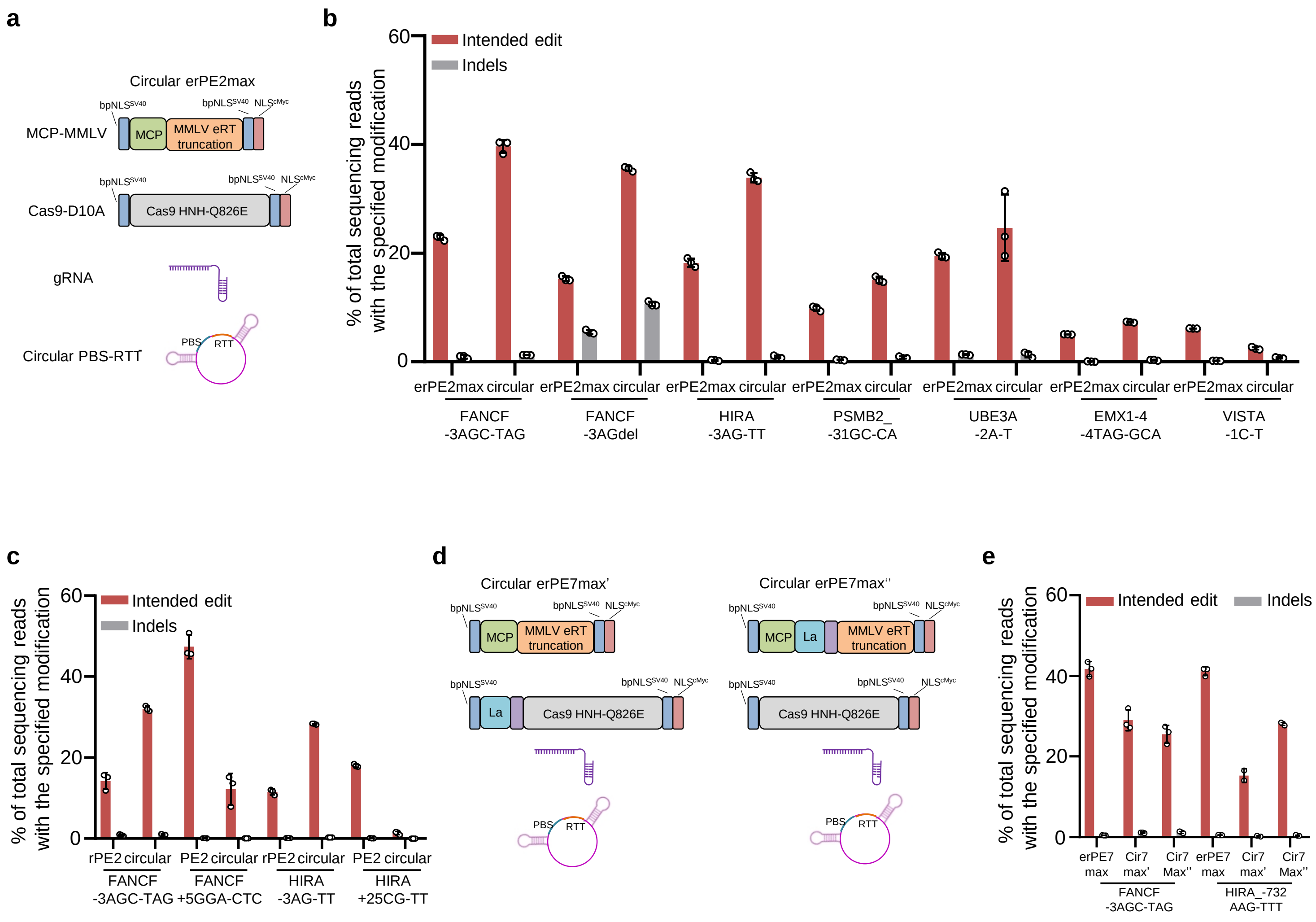

Fig S8. Verification of circular erPEmax in human cells

(a) Schematic of circular erPE2max. It was composed with MS2-coat protein (MCP)-MMLV eRT (Q291I+D339E), Cas9-D10A, gRNA and circular PBS-RTTs with MS2 hairpin.

(b) Editing efficiency (red bars) and indel frequency (gray bars) between erPE2max and its circular pattern across 7 genomic loci in HKE293T cells. Data are presented as means ± SD from n = 3 independent biological replicates.

(c) Comparison of editing efficiency (red bars) and indel frequency (gray bars) between rPE2/PE2 and their circular pattern at 2 genomic loci. Data are presented as means ± SD from n = 3 independent biological replicates.

(d) Schematic of circular erPE7max variants. It was composed with MS2-coat protein (MCP)-MMLV eRT (Q291I+D339E), Cas9-D10A, gRNA and circular PBS-RTTs with MS2 hairpin. The La was set in Cas9 (left) and MMLV eRT (right).

(e) Comparison of editing efficiency (red bars) and indel frequency (gray bars) across circular erPE7max variants at 2 genomic loci in HEK293T cells. Data are presented as means ± SD from n = 3 independent biological replicates.
