# Supplementary material for: Prime editor with rational design and AI-driven optimization for reverse editing window and enhanced fidelity
Source: Nat Commun. 2025 Jun 3;16:5144. doi: 10.1038/s41467-025-60495-w (PMC12134370; doi:10.1038/s41467-025-60495-w)
Supplement: Supplementary file 2 — Description of Additional Supplementary Files [file 41467_2025_60495_MOESM2_ESM.pdf]

Description of Additional Supplementary Files

File Name: Supplementary Data 1
Description: Scores of PSSM, MSA-1b, esm-1v and esm-if1 for MMLV and HNH.

File Name: Supplementary Data 2
Description: Primers of constructing sgRNA.

File Name: Supplementary Data 3
Description: Detailed information for pegRNA/rpegRNA in all figures and their deep sequencing olgios.

File Name: Supplementary Data 4
Description: Deep sequencing olgios of sgRNA On-target and Off-target site.
