# Supplementary material for: Prime editor with rational design and AI-driven optimization for reverse editing window and enhanced fidelity
Source: Nat Commun. 2025 Jun 3;16:5144. doi: 10.1038/s41467-025-60495-w (PMC12134370; doi:10.1038/s41467-025-60495-w)
Supplement: Supplementary file 7 — Reporting Summary [file 41467_2025_60495_MOESM7_ESM.pdf]

nature portfolio

Corresponding author(s): Changhao Bi

Last updated by author(s): May 8, 2025

Reporting Summary

Nature Portfolio wishes to improve the reproducibility of the work that we publish. This form provides structure for consistency and transparency in reporting. For further information on Nature Portfolio policies, see our Editorial Policies and the Editorial Policy Checklist.

Statistics

For all statistical analyses, confirm that the following items are present in the figure legend, table legend, main text, or Methods section.

n/a	Confirmed	
☐	☒	The exact sample size (*n*) for each experimental group/condition, given as a discrete number and unit of measurement
☒	☐	A statement on whether measurements were taken from distinct samples or whether the same sample was measured repeatedly
☒	☐	The statistical test(s) used AND whether they are one- or two-sided<br>*Only common tests should be described solely by name; describe more complex techniques in the Methods section.*
☒	☐	A description of all covariates tested
☒	☐	A description of any assumptions or corrections, such as tests of normality and adjustment for multiple comparisons
☐	☒	A full description of the statistical parameters including central tendency (e.g. means) or other basic estimates (e.g. regression coefficient) AND variation (e.g. standard deviation) or associated estimates of uncertainty (e.g. confidence intervals)
☐	☒	For null hypothesis testing, the test statistic (e.g. *F*, *t*, *r*) with confidence intervals, effect sizes, degrees of freedom and *P* value noted<br>*Give P values as exact values whenever suitable.*
☒	☐	For Bayesian analysis, information on the choice of priors and Markov chain Monte Carlo settings
☒	☐	For hierarchical and complex designs, identification of the appropriate level for tests and full reporting of outcomes
☒	☐	Estimates of effect sizes (e.g. Cohen's *d*, Pearson's *r*), indicating how they were calculated

Our web collection on statistics for biologists contains articles on many of the points above.

Software and code

Policy information about availability of computer code

Data collection	illumina Hiseq Control Software (2.7) was used to collect the high-throughput sequencing data; Alphafold 2 was used to collect protein structure information
Data analysis	CRISPResso2, esm-1v, esm-mas-1b, esm-if1, PSSM

For manuscripts utilizing custom algorithms or software that are central to the research but not yet described in published literature, software must be made available to editors and reviewers. We strongly encourage code deposition in a community repository (e.g. GitHub). See the Nature Portfolio guidelines for submitting code & software for further information.

Data

Policy information about availability of data

All manuscripts must include a data availability statement. This statement should provide the following information, where applicable:

- Accession codes, unique identifiers, or web links for publicly available datasets
- A description of any restrictions on data availability
- For clinical datasets or third party data, please ensure that the statement adheres to our policy

There is no restriction on the data associated with this study. HTS data generated in this study have been deposited in the NCBI Sequence Read Archive database under accession code PRJNA1099390 (https://www.ncbi.nlm.nih.gov/bioproject/1099390). Source data are provided with this paper.

Research involving human participants, their data, or biological material

Policy information about studies with human participants or human data. See also policy information about sex, gender (identity/presentation), and sexual orientation and race, ethnicity and racism.

Reporting on sex and gender	Sex was not considered as the study was based on human cell lines and organoid models, which are not affected by sex-specific physiological factors.
Reporting on race, ethnicity, or other socially relevant groupings	PBMCs and primary tissues were chosen without regard to sex, gender, ethnicity or race.
Population characteristics	PBMCs and primary tissues were chosen without regard to sex, gender, ethnicity or race.
Recruitment	Primary tissues was obtained from patient that was diagnosed with pancreatic cancer; PBMCs were obtained from healthy donors.
Ethics oversight	The study complied with all the ethical regulations for work with patients. The study was approved by Ethics Committee of Tianjin Medical University Cancer Institute and Hospital and written informed consent was obtained from all patients.

Note that full information on the approval of the study protocol must also be provided in the manuscript.

Field-specific reporting

Please select the one below that is the best fit for your research. If you are not sure, read the appropriate sections before making your selection.

☒ Life sciences ☐ Behavioural & social sciences ☐ Ecological, evolutionary & environmental sciences

For a reference copy of the document with all sections, see nature.com/documents/nr-reporting-summary-flat.pdf

Life sciences study design

All studies must disclose on these points even when the disclosure is negative.

Sample size	The number of replicates was selected based on common practice in similar studies and prior publications to ensure reproducibility and reliability of results.
Data exclusions	No data or samples were excluded from the analyses. All collected data were included in the final analysis.
Replication	All experiments were independently performed in biological triplicates, and similar results were consistently observed across replicates.
Randomization	Experimental groups were randomly assigned. As the study was conducted in cultured cell lines under controlled conditions, randomization was sufficient to minimize technical variability.
Blinding	Blinding was not relevant to this study, as all experiments were conducted using in vitro gene-edited cell lines with objective and quantifiable measurements, minimizing the potential for observer bias.

Reporting for specific materials, systems and methods

We require information from authors about some types of materials, experimental systems and methods used in many studies. Here, indicate whether each material, system or method listed is relevant to your study. If you are not sure if a list item applies to your research, read the appropriate section before selecting a response.

Materials & experimental systems

n/a	Involved in the study	
☒	☐	Antibodies
☐	☒	Eukaryotic cell lines
☒	☐	Palaeontology and archaeology
☒	☐	Animals and other organisms
☒	☐	Clinical data
☒	☐	Dual use research of concern
☒	☐	Plants

Methods

n/a	Involved in the study	
☒	☐	ChIP-seq
☒	☐	Flow cytometry
☒	☐	MRI-based neuroimaging

Eukaryotic cell lines

Policy information about cell lines and Sex and Gender in Research

Cell line source(s)	HEK293T (ATCC® CRL-3216™), HeLa (ATCC® CCL-2™), Jurkat (ATCC® TIB-152™) and HepG2 (ATCC® HB-8065™) cell lines were obtained from the American Type Culture Collection (ATCC, Manassas, VA, USA).
Authentication	No cell lines were authenticated.
Mycoplasma contamination	Cells were not tested for mycoplasma contamination.
Commonly misidentified lines (See ICLAC register)	No cell lines were used that are in the ICLAC register.

Plants

Seed stocks	Not applicable
Novel plant genotypes	Not applicable
Authentication	Not applicable
